# Integrated Analysis of Hub Genes and MicroRNAs in Human Placental Tissues from *In Vitro* Fertilization-Embryo Transfer

**DOI:** 10.3389/fendo.2021.774997

**Published:** 2021-11-11

**Authors:** Shuheng Yang, Wei Zheng, Chen Yang, Ruowen Zu, Shiyu Ran, Huan Wu, Mingkun Mu, Simin Sun, Nana Zhang, Rick F. Thorne, Yichun Guan

**Affiliations:** ^1^ Center for Reproductive Medicine, The Third Affiliated Hospital of Zhengzhou University, Zhengzhou, China; ^2^ Translational Research Institute, Henan Provincial People's Hospital, Zhengzhou, China; ^3^ Academy of Medical Sciences, Zhengzhou University, Zhengzhou, China

**Keywords:** *in vitro* fertilization-embryo transfer, placenta, differentially expressed genes, MiRNA-mRNA, next-generation sequencing

## Abstract

**Objective:**

Supraphysiological hormone exposure, *in vitro* culture and embryo transfer throughout the *in vitro* fertilization-embryo transfer (IVF-ET) procedures may affect placental development. The present study aimed to identify differences in genomic expression profiles between IVF-ET and naturally conceived placentals and to use this as a basis for understanding the underlying effects of IVF-ET on placental function.

**Methods:**

Full-term human placental tissues were subjected to next-generation sequencing to determine differentially expressed miRNAs (DEmiRs) and genes (DEGs) between uncomplicated IVF-ET assisted and naturally conceived pregnancies. Gene ontology (GO) enrichment analysis and transcription factor enrichment analysis were used for DEmiRs. MiRNA-mRNA interaction and protein-protein interaction (PPI) networks were constructed. In addition, hub genes were obtained by using the STRING database and Cytoscape. DEGs were analyzed using GO and Kyoto Encyclopedia of Genes and Genomes (KEGG) pathway analysis. Differentially expressed miRNAs were validated through qRT-PCR.

**Results:**

Compared against natural pregnancies, 12 DEmiRs and 258 DEGs were identified in IVF-ET placental tissues. In a validation cohort, it was confirmed that hsa-miR-204-5p, hsa-miR-1269a, and hsa-miR-941 were downregulation, while hsa-miR-4286, hsa-miR-31-5p and hsa-miR-125b-5p were upregulation in IVF-ET placentas. Functional analysis suggested that these differentially expressed genes were significantly enriched in angiogenesis, pregnancy, PI3K-Akt and Ras signaling pathways. The miRNA-mRNA regulatory network revealed the contribution of 10 miRNAs and 109 mRNAs while EGFR was the most highly connected gene among ten hub genes in the PPI network.

**Conclusion:**

Even in uncomplicated IVF-ET pregnancies, differences exist in the placental transcriptome relative to natural pregnancies. Many of the differentially expressed genes in IVF-ET are involved in essential placental functions, and moreover, they provide a ready resource of molecular markers to assess the association between placental function and safety in IVF-ET offspring.

## Introduction

*In vitro* fertilization-embryo transfer (IVF-ET) is the main method of assisted reproductive technology (ART), and can improve the success rates of infertility treatment (1, 2). The use of this procedure has been steadily increasing worldwide and for example, in China, the average number of ART treatments performed now exceeds 700,000 annually (3). The vast majority of children successfully delivered through ART are healthy (4). Nonetheless, there are increased risks of several pregnancy-related complications, including gestational diabetes (5), pre-eclampsia (6), placenta previa (7), abnormal placental growth, preterm delivery (8), and low birth weight (9). Some reports have suggested that the adverse perinatal outcomes occur due to IVF-ET procedures such as supraphysiological estrogen level during stimulation, *in vitro* culture, and microscopic manipulation (10, 11). However, the underlying causes of IVF-ET-associated complications are largely unknown, although many conceptually appear related to placental vascular complications and the resulting effects on fetal development.

MicroRNAs (miRNAs) represent a major class of non-coding RNAs consisting of single-stranded RNAs of approximately 18-25 nucleotides in length (12). They function as negative gene regulators by binding to the 3’ UTR of messenger RNAs (mRNAs), to either prevent protein translation or to direct the mRNA towards degradation (13). The placenta expresses many ubiquitous as well as specific miRNAs and a growing body of evidence proposes that miRNAs function as important regulators of placental development (14). Here, various miRNAs have been shown to control the differentiation, replication, apoptosis, invasion/migration and angiogenesis of trophoblasts, indicating the widespread contribution of miRNAs to the placental growth (15, 16). For example, miR-346 and miR-582-3p down-regulate the expression of endocrine gland-derived vascular endothelial growth factor (*EG-VEGF*) and inhibit trophoblast cell invasion and migration (17). miR-191 inhibits angiogenesis by activating the nuclear factor-κB (NF-κB) signaling pathway (18). It was also found that miR-29b inhibited trophoblast invasion and angiogenesis by suppressing vascular endothelial growth factor (*VEGF*) expression (19). In addition, animal experiments in mice revealed that miR-450a-3p played a role in inhibiting cell proliferation, promoting apoptosis and interfering with embryonic development through regulating the target gene *Bub1* (20). However, the impact of specific microRNAs in IVF-ET placental tissue, and their potential impact on related gene regulatory networks has to date been poorly investigated.

This study aimed to understand the differences in placental genomic expression profiles comparing IVF-ET and natural pregnancy-derived placentas, and the link between IVF-ET manipulation and placental structure and function. We used next-generation sequencing techniques to analyze of miRNA and mRNA expression profiles in IVF-ET and natural gestational placental tissue. Based on co-expression analysis and online prediction. we established a miRNA-mRNA regulatory network comprising 10 miRNAs and 109 mRNAs together with a PPI network comprised of ten hub genes. Furthermore, we verified that miR-204-5p, miR-1269a and miR-941 were downregulated in IVF-ET placentals thereby proposing these as key regulators involved in the effects of IVF-ET on placental development and function. Moreover, our study constitutes a verified resource for enabling further investigation into the transcriptomic and mechanistic differences between IVF-ET and naturally conceived pregnancies with the goal of providing new targets to assess the safety of IVF-ET in the clinic.

## Materials and Methods

### Tissue Collection and Ethics

Placental tissue samples were collected from women who underwent caesarean deliveries after IVF-ET assisted (n=3) or natural conceived (n=3) pregnancies. Inclusion criteria included full-term singleton delivery after IVF-ET, with age between 20 and 35 years, 37-42 gestational weeks, infant birth weight between 2500 g and 4000 g, and uncomplicated pregnancies. Three strictly matched natural pregnancies were selected as controls with matching parameters: delivery, maternal age, parity, and gestational duration (**Table 1**). A validation cohort of 8 uncomplicated IVF-ET and 8 normal conception patients were similarly collected (**Table 2**). We used an equal number of male and female placentals for both discovery and validation cohorts and selected tissue from the middle placenta throughout to minimize sampling bias. All placental tissues were rinsed extensively with ice cold PBS and stored at -80°C until later RNA extraction. Tissue collection was approved by the Ethics Committee of the Third Affiliated Hospital of Zhengzhou University with written informed consent provided by all patients prior to sample collection.

**Table 1:** Clinical characteristics of IVF-ET and controls for high-throughput sequencing.

Cases	Age (years)	Gravidity	Parity	Gestational week at delivery	Mode of delivery	Sex of the baby	Birth weight(g)	Weight of placenta(g)	Baby/placenta weight
Control 1	27	1	0	39	Cesarean	Female	3150	500	6.30
Control 2	28	1	0	40.29	Cesarean	Male	3850	630	6.11
Control 3	21	1	0	39.71	Cesarean	Male	3500	510	6.86
IVF-ET 1	31	2	0	38.14	Cesarean	Male	3000	540	5.56
IVF-ET 2	33	1	0	38.57	Cesarean	Female	2800	480	5.83
IVF-ET 3	29	1	0	38.14	Cesarean	Male	3100	540	5.74

**Table 2:** Clinical characteristics of IVF-ET and controls.

Clinical features	IVF-ET (n = 8)	Control (n = 8)	*P* value
Maternal age (years)	31.13 ± 2.58	28.63 ± 2.67	0.078
Gestational week at delivery	39.41 ± 0.69	39.43 ± 0.86	0.967
Mode of delivery	Cesarean	Cesarean	
Birth weight(g)	3512.50 ± 410.64	3686.25 ± 302.04	0.351
Infant sex			
Female	4	4	
Male	4	4	
Baby/placenta weight	5.94 ± 0.46	5.69 ± 0.70	0.387

Data are presented as mean ± SD. t-test. IVF-ET, in vitro fertilization-embryo transfer.

### RNA Extraction and Sequencing

Total RNA was isolated from placental tissue using the mirVana RNA Isolation Kit (Cat #. AM1561, Austin TX, US) according to the manufacturer’s instructions. RNA concentration and integrity were then verified using an Agilent Bioanalyzer 2100 (Agilent technologies Santa Clara, US) before subjecting the samples to library preparation and sequencing. The concentration and size of the constructed libraries were measured using a Qubit^®^ 2.0 Fluorometer (Life Technologies, USA) and Agilent 2100 Bioanalyzer, respectively. The samples were prepared according to the HiSeq 2500 User Guide, and the flow cell with the cluster was loaded on the Illumina HiSeq 2500 (50-bp single-end FASTQ reads). The number of sequencing reads per sample was at least 10M and the proportion of bases with mass greater than 20 was greater than 95%, and the quality control meets the requirements of data analysis.

### Differential Expression Analysis

The DESeq2 package of R version 3.5.2 (http://www.r-project.org) was used to define differentially expressed miRNAs (DEmiRs) and genes (DEGs) with the Bayesian method used to correct batch effects. MiRNAs and mRNAs with statistical significance between the IVF-ET and control groups were selected according to the threshold criterion of fold change (FC) >1.5 and *P* < 0.05. Volcano maps were created in the R studio using the plot packages to illustrate the differential expression of DEmiRs and DEGs.

### GO Enrichment Analysis for the Targets of Transcription Factors

DEmiRs were uploaded to FunRich software to screen for upstream transcription factors, which is primarily used for functional enrichment and interaction network analysis of genes and proteins, as well as enrichment targets for transcription factor pathways (21). For interaction network analysis between miRNAs, gene/mRNA, and transcription factors, gene ontogeny (GO) enrichment analysis was also used (22, 23).

### Construction of the miRNA-mRNA Regulatory Networks

The miRWalk V2.0, StarBase and TargetScan databases were used to predict the target mRNAs of the miRNAs identified as DEmiRs. Subsequently, the predicted DEGs were matched with the experimentally determined DEGs to develop miRNA-mRNA regulatory networks with visualization using Cytoscape software (http://www.cytoscape.org/) (24). All node degrees, proximity and presence of the regulatory network were simultaneously computed.

### GO and KEGG Enrichment Analyses

For gene ontology (GO) and Kyoto Encyclopedia of Genes and Genomes (KEGG) pathway analysis of DEGs, we used the Database for Annotation, Visualization and Integrated Discovery (DAVID) and used ggplot2 packages in the R studio to identify significantly altered biological processes (BPs), cellular components (CCs), molecular functions (MFs) and pathways associated with DEGs (*P* < 0.05).

### Protein-Protein Interaction (PPI) Network Analysis and Hub Gene Identification

Differentially expressed gene data was uploaded to the STRING database (http://www.string-db.org/) (25). Interactions with a composite score of >0.4 were considered significant. The target genes in the PPI network act as nodes and the line from two nodes indicates relevant interactions. Cytoscape software was used to visualize the PPI network. We screened the top 10 genes with the highest degree of correlation to the others as hub genes with the CytoHubba plugin of Cytoscape (26).

### Quantitative Real-Time Polymerase Chain Reaction (qRT-PCR)

One μg total RNA was reverse transcribed into cDNA using the ReverTra Ace qPCR RT Kit (Toyobo, Japan) according to the manufacturers’ instructions. Specific primers were used to synthesize the cDNA of miRNAs. qRT-PCR reactions were performed with the indicated primers (**Table 3**) in triplicate 20 μL reactions using the SYBR Green Realtime PCR Master Mix (Toyobo, Japan) on a StepOnePlus™ Real-Time PCR System. Cycling conditions were as follows: 95°C for 60 seconds, 40 cycles of 95°C for 15 seconds, 60°C for 15 seconds, and 72°C for 45 seconds. The results were normalized to U6 and the relative changes calculated using the 2^−ΔΔCt^ method (27).

**Table 3:** Oligonucleotides used in this study.

Primer sets name	Reverse transcriptase primer (5′ to 3′)	Real-time quantitative PCR primer (5′ to 3′)
U6	AACGCTTCACGAATTTGCGT	F:CTCGCTTCGGCAGCACA
		R: AACGCTTCACGAATTTGCGT
has-miR-204-5p	GTCGTATCCAGTGCAGGGTCCGAGGTAT	F: CGCGTTCCCTTTGTCATCCT
	TCGCACTGGATACGACAGGCAT	R:AGTGCAGGGTCCGAGGTATT
has-miR-1269a	GTCGTATCCAGTGCAGGGTCCGAGGTAT	F: CGCTGGACTGAGCCGTG
	TCGCACTGGATACGACCCAGTA	R: AGTGCAGGGTCCGAGGTATT
has-miR-941	GTCGTATCCAGTGCAGGGTCCGAGGTAT	F: CACCCGGCTGTGTGCAC
	TCGCACTGGATACGACGCACAT	R: AGTGCAGGGTCCGAGGTATT
has-miR-4286	GTCGTATCCAGTGCAGGGTCCGAGGTAT	F: GCGCGACCCCACTCCT
	TCGCACTGGATACGACGGTACC	R: AGTGCAGGGTCCGAGGTATT
has-miR-31-5p	GTCGTATCCAGTGCAGGGTCCGAGGTAT	F: GCGAGGCAAGATGCTGGC
	TCGCACTGGATACGACAGCTAT	R: AGTGCAGGGTCCGAGGTATT
has-miR-125b-5p	GTCGTATCCAGTGCAGGGTCCGAGGTAT	F: CGCGTCCCTGAGACCCTAAC
	TCGCACTGGATACGACTCACAA	R: AGTGCAGGGTCCGAGGTATT

### Statistical Analysis

Data were presented as means ± standard deviations (SD) for quantitative variables and the Student’s *t*-test used to assess differences between groups. Otherwise, for discrete variables the Mann-Whitney *U*-test was used. A value of *P* < 0.05 was regarded as statistically significant. SPSS 21.0 and GraphPad Prism 6.0 were used for analysis.

## RESULTS

### Identification of Differentially Regulated Genes in IVF-ET Placentals

**Figure 1:**
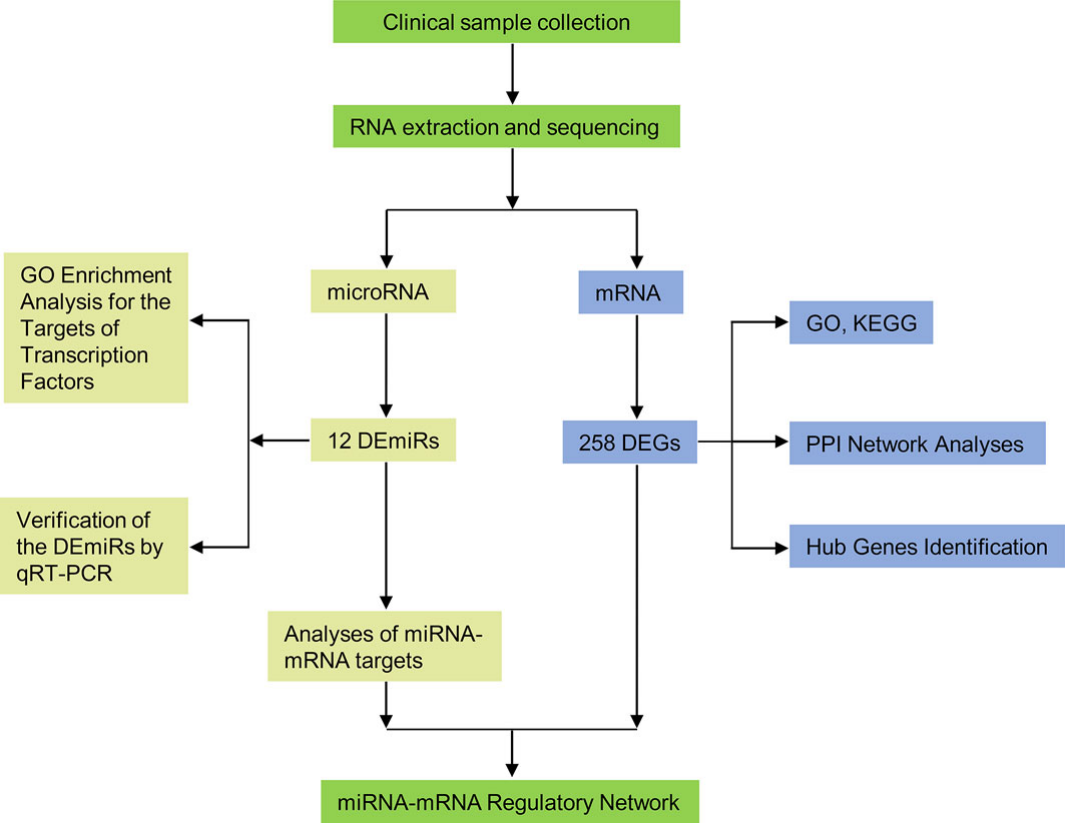
Flow diagram of the study design. DEmiRs, differentially expressed miRNAs; DEGs, differentially expressed genes; GO, Gene Ontology; KEGG, Kyoto Encyclopedia of Genes and Genomes; PPI, protein-protein interaction.

**Figure 2:**
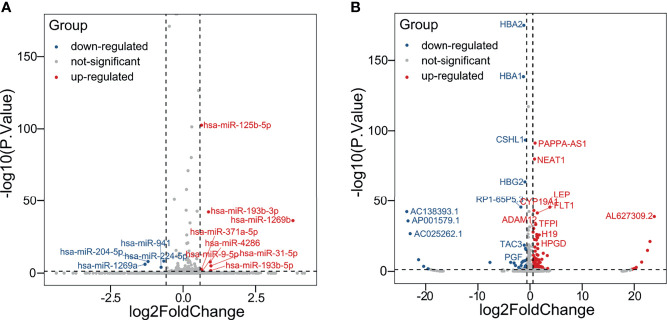
Identification of DEmiRs and DEGs. Volcano plots illustrating **(A)** DEmiRs and **(B)** DEGs in placental tissues determined by comparing IVF-ET (n = 3) with normal conception (n = 3) pregnancies. Differential expression thresholds employed for deriving DEmiRs and DEGs involved fold change (FC) >1.5 and *P* < 0.05. Red and blue points represent significantly upregulated or downregulated miRNAs/mRNAs, respectively.

The schema of the overall study and analysis approach is presented in **Figure 1**. High throughput sequencing analysis was performed to analyze the expression profiles of miRNAs and mRNAs in placentals from uncomplicated full-term IVF-ET and natural conception pregnancies. From these data we identified a total of 12 differentially expressed miRNAs (DEmiRs; **Figure 2A**) including 4 downregulated miRNAs: hsa-miR-1269a, hsa-miR-204-5p, hsa-miR-224-5p and hsa-miR-941, and 8 upregulated miRNAs: hsa-miR-1269b, hsa-miR-125b-5p, hsa-miR-193b-3p, hsa-miR-193b-5p, hsa-miR-31-5p, hsa-miR-371a-5p, hsa-miR-4286 and hsa-miR-9-5p (**Table 4**). We also identified 258 differentially expressed mRNAs consisting of 52 downregulated and 206 upregulated DEGs (**Figure 2B** and **Supplementary Table SI**).

**Table 4:** The 12 differentially expressed miRNAs (DEmiRs) in IVF-ET.

Symbol	*P* Value	logFC	Up/Down
hsa-miR-941	<0.001	-0.758138251	Down
hsa-miR-9-5p	0.047	0.659018502	Up
hsa-miR-4286	<0.001	0.938226403	Up
hsa-miR-371a-5p	0.003	0.633507315	Up
hsa-miR-31-5p	<0.001	0.962107998	Up
hsa-miR-224-5p	<0.001	-0.664686886	Down
hsa-miR-204-5p	<0.001	-1.207550612	Down
hsa-miR-193b-5p	0.020	0.954586891	Up
hsa-miR-193b-3p	<0.001	0.878208945	Up
hsa-miR-1269b	<0.001	3.787867629	Up
hsa-miR-1269a	<0.001	-1.314083333	Down
hsa-miR-125b-5p	<0.001	0.639432322	Up

IVF-ET, in vitro fertilization-embryo transfer; FC, fold change.

### Verification of the DEmiRs by qRT-PCR in Placental Tissues

**Figure 3:**
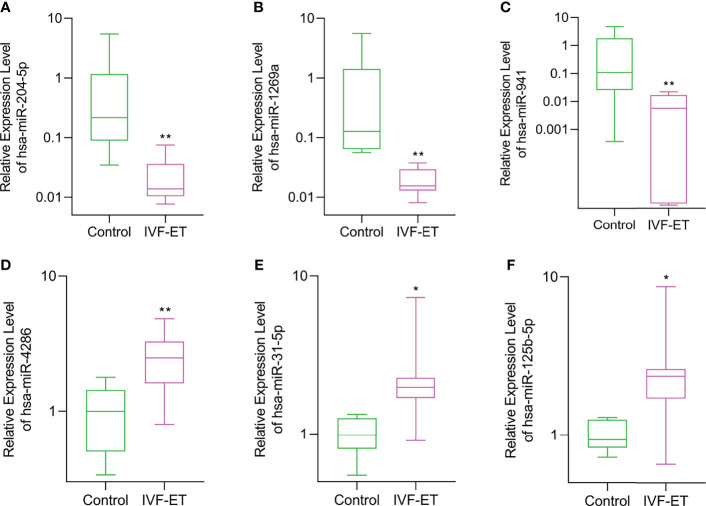
Validation of differential expression of randomly selected DEmiRs in IVF-ET *versus* control placental tissues. The relative expression levels of **(A)** hsa-miR-204-5p, **(B)** hsa-miR-1269a, **(C)** hsa-miR-941, **(D)** hsa-miR-4286, **(E)** hsa-miR-31-5p and **(F)** hsa-miR-125b-5p were measured in an independent cohort of placental tissues from uncomplicated IVF-ET (n = 8) and natural conceived pregnancies (n = 8). Box and whisker plot with relative expression plotted in log scale showing the range (whiskers), first and third quartiles (boxes) and median values. ^*^ *P* < 0.05, ^**^ *P* < 0.01.

To ensure the veracity of the high throughput sequencing analysis, it was necessary to validate our findings using alternative methodology and samples. On the basis of subsequent bioinformatics (see below), we selected 6 of the 12 DEmiRs (3 downregulated and 3 upregulated, respectively) and analyzed their expression using qRT-PCR in a validation cohort of 8 IVF-ET and 8 normal conception placentas. Instructively, we found that the relative expression levels of hsa-miR-204-5p, hsa-miR-1269a, and hsa-miR-941 were significantly downregulated whereas hsa-miR-4286, hsa-miR-31-5p and hsa-miR-125b-5p were all upregulated, respectively, in IVF-ET compared to the control placentas (**Figures 3A–F**). These results suggest that the dysregulation of these miRNAs commonly occurs in IVF-ET pregnancies but further verification will be required to support the general veracity of the global sequencing data and analysis of the study.

### Transcription Factor Enrichment and GO Enrichment Analysis

**Figure 4:**
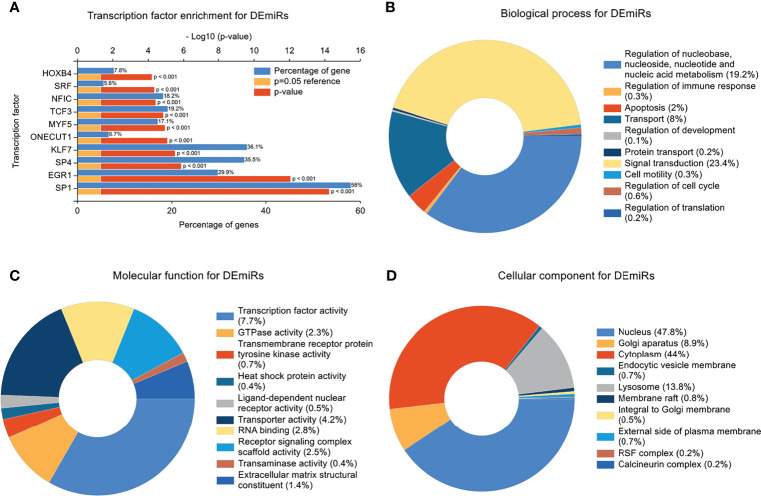
Transcription Factor Enrichment and GO Enrichment Analysis. **(A)** Transcription factors (TF) of the differentially expressed miRNAs (DEmiRs) from FunRich. Blue bars, orange bars, and red bars represent percentage of predicted genes, reference of P=0.05, and *p* value, respectively. **(B)** mRNAs involved in biological process terms for DEmiRs. **(C)** mRNAs involved in molecular function terms for DEmiRs. **(D)** mRNAs involved in cellular component terms for DEmiRs.

Transcription factors often represent the critical final step in signal transduction pathways. To investigate the enrichment of transcription factor targets likely associated with the genetic landscape of the IVF-ET placenta, we filtered out the top 10 transcription factors most closely linked to the DEmiRs. In deceasing probability order, this analysis identified SP1, EGR1, SP4, KLF7, ONECUT1, MYF5, TCF3, NFIC, SRF, and HOXB4 (**Figure 4A**). It indicates a regulatory interaction between these transcription factors and DEmiRs. Intriguingly, SP1 was apparently able to regulate majority of the DEmiRs.

In concert with these findings, GO enrichment analysis indicated that the top 5 biological progress (BP) terms with the most enriched targets of the DEmiRs involved signal transduction; regulation of nucleobase, nucleoside, nucleotide and nucleic acid metabolism, transport, apoptosis, and regulation of immune response (**Figure 4B**). GO enrichment terms associated with molecular function (MF) indicated most of the genes were involved in transcription factor activity, transporter activity, RNA binding, Receptor signaling complex scaffold activity, and GTPase activity (**Figure 4C**). The top 5 enriched cellular component (CC) terms were nucleus, cytoplasm, Golgi apparatus, lysosome and membrane raft (**Figure 4D**).

### Construction of miRNA-mRNA Regulatory Networks

**Figure 5:**
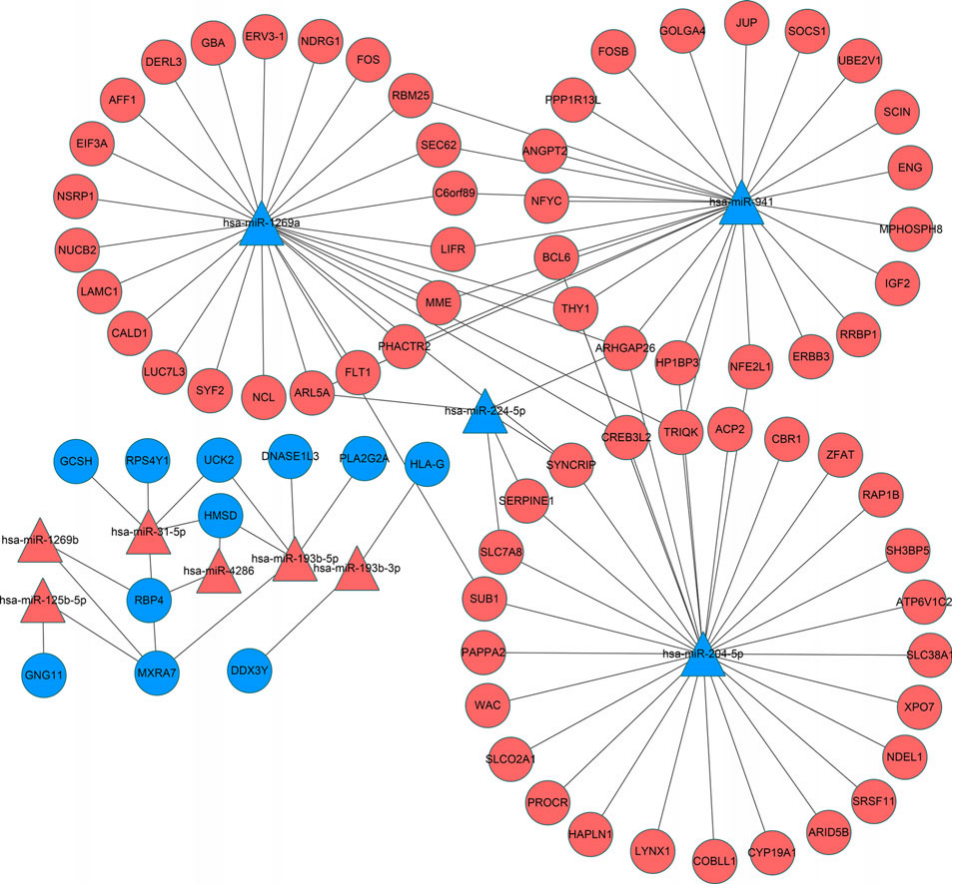
Interaction networks of miRNA and target DEGs in IVF-ET placenta tissues. microRNAs are represented by triangles and mRNAs are represented by circles. Red indicates genes with up-regulated expression and blue indicates genes with down-regulated expression. miRNA, microRNA; mRNA, messenger RNA; IVF-ET, *in vitro* fertilization-embryo transfer.

Reliable identification of miRNA targets is still an imprecise process, but it is widely appreciated that predictions can be improved using the outputs of multiple algorithms (28). Consequently, we employed three databases (miRWalk V2.0, StarBase and TargetScan) to analyze the potential impact of the DEmiRs on the placental transcriptome. Screening miRNA targets based on the overlapping results of the three databases and the intersection with DEGs. This analysis yielded paired interactions between 109 DEGs with 10 of the 12 identified DEmiRs. The network of miRNA-mRNA interactions was visualized in Cytoscape (**Figure 5**) and the target genes of the DEmiRs are listed in **Table 5**. Notably, among these, the four downregulated DEmiRs, particularly hsa-miR-204-5p, hsa-miR-1269a and hsa-miR-941, formed the most extensive interactive network with multiple gene targets while the upregulated DEmiRs aligned with a more discrete set of target genes.

**Table 5:** The miRNA-mRNA network.

Symbol	Up/Down	Count	Target mRNA
hsa-miR-941	Down	28	RBM25, MPHOSPH8, SOCS1, SEC62, HP1BP3, JUP, NFE2L1, THY1, FLT1, GOLGA4, TRIQK, C6orf89, NFYC, PPP1R13L, ARHGAP26, IGF2, BCL6, LIFR, ANGPT2, FOSB, SCIN, PHACTR2, RRBP1, ARL5A, MME, ERBB3, UBE2V1, ENG
hsa-miR-4286	Up	2	RBP4, HMSD
hsa-miR-31-5p	Up	5	GCSH, UCK2, HMSD, MXRA7, RPS4Y1
hsa-miR-224-5p	Down	5	SYNCRIP, ARHGAP26, ARL5A, SERPINE1, SLC7A8
hsa-miR-204-5p	Down	30	PAPPA2, WAC, SUB1, PROCR, SLCO2A1, HAPLN1, HP1BP3, LYNX1, NFE2L1, THY1, ACP2, CBR1, COBLL1, SLC7A8, CREB3L2, ARID5B, SRSF11, TRIQK, SYNCRIP, ARHGAP26, BCL6, XPO7, SLC38A1, ATP6V1C2, ZFAT, NDEL1, SH3BP5, RAP1B, SERPINE1, CYP19A1
hsa-miR-193b-5p	Up	5	UCK2, DNASE1L3, HMSD, MXRA7, PLA2G2A
hsa-miR-193b-3p	Up	2	HLA-G, DDX3Y
hsa-miR-1269b	Up	2	RBP4, MXRA7
hsa-miR-1269a	Down	28	RBM25, SYF2, SUB1, NCL, FOS, CALD1, THY1, SEC62, LAMC1, AFF1, LUC7L3, NUCB2, EIF3A, C6orf89, NSRP1, CREB3L2, TRIQK, FLT1, SYNCRIP, ARHGAP26, NDRG1, LIFR, PHACTR2, ARL5A, ERV3-1, DERL3, MME, GBA
hsa-miR-125b-5p	Up	2	GNG11, MXRA7

### Functional Enrichment Analysis of the DEGs

**Figure 6:**
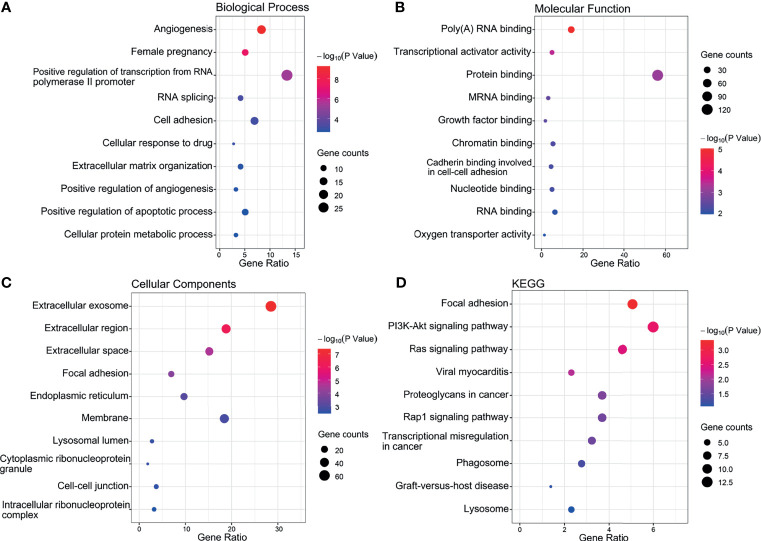
Top 10 significant enrichment GO and KEGG terms of DEGs. **(A)** BP, biological process. **(B)** MF, molecular function. **(C)** CC, cellular component. **(D)** KEGG, Kyoto Encyclopedia of Genes and Genomes.

Independent of the preceding analysis, we utilized ggplot2 and enrichment analysis to profile GO annotations and KEGG pathways associated with the differentially expressed genes. These predictions would allow an improved understanding of the functional impact of the genes dysregulated in IVF-ET. As illustrated, the top 10 enriched GO and KEGG terms are presented in categories of biological processes (BPs), cellular components (CCs), molecular functions (MFs) and defined KEGG pathway identifiers (**Figure 6**).

Notably, this analysis revealed significant enrichment for BP entries aligned with angiogenesis, pregnancy, cell adhesion, positive regulation of transcription from the RNA polymerase II promoter and positive regulation of angiogenesis (**Figure 6A**). Furthermore, the protein binding, poly (A) RNA binding and transcriptional activator activity accounted for the majority of MF terms (**Figure 6B**) while the most enriched CCs were extracellular exosome, membrane, and extracellular region (**Figure 6C**). The top 10 most highly enriched KEGG classifications included the PI3K-Akt and Ras signaling pathways along with focal adhesion (**Figure 6D**).

### Construction of Protein-Protein Interaction (PPI) Networks

**Figure 7:**
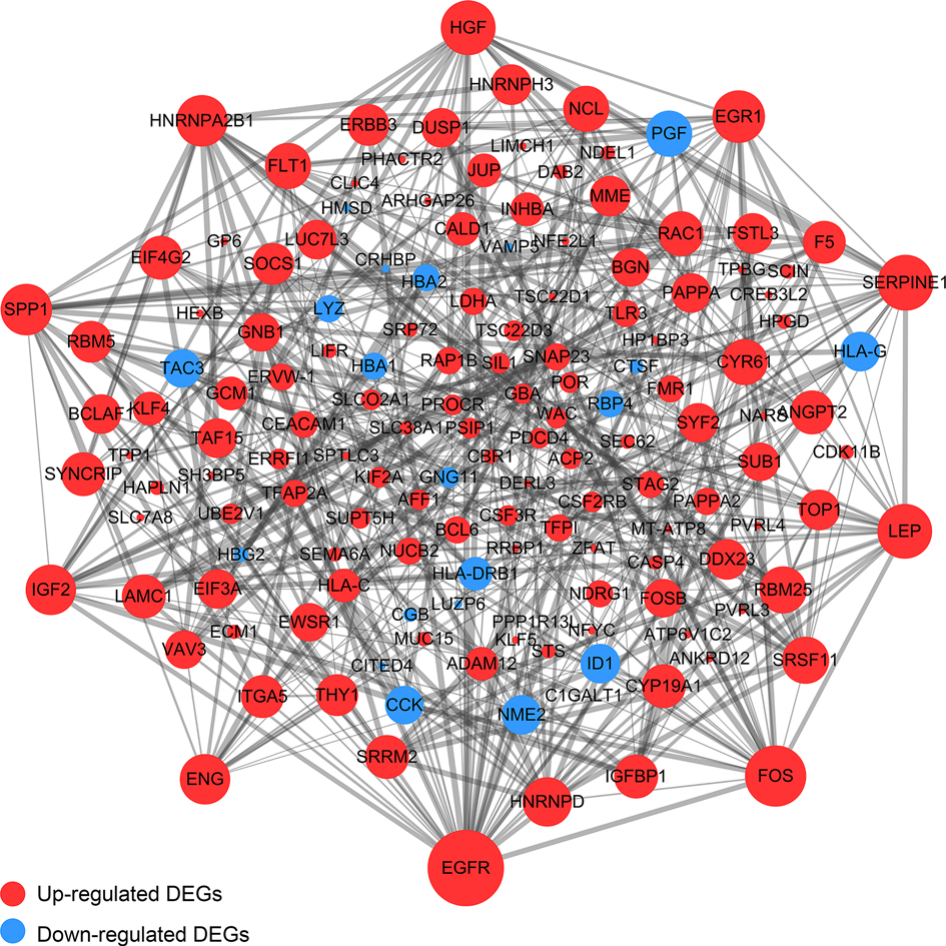
The PPI Network of DEGs. The upregulated genes were exhibited by the red color, while the blue color exhibited the downregulated genes. Node size is proportional to the degree of the node itself. Edge width is proportional to the combined degree between genes.

Using the DEGs we next built a PPI network using the online STRING database and tools in Cytoscape. The network was mapped to a limit of 148 DEGs (**Figure 7**). Node size is proportional to the degree of the node itself. Edge width is proportional to the combined degree between genes. We assessed their degree of connectivity and identified 10 hub genes, and the genes are listed in **Table 6**.

**Table 6:** The top 10 genes in the network are ranked in order of degree.

Rank	Symbol	Score
1	EGFR	40
2	FOS	27
3	SERPINE1	21
3	LEP	21
5	HGF	19
5	EGR1	19
7	SPP1	18
8	HNRNPA2B1	17
9	IGF2	16
9	ENG	16

### Biological Analysis of the Hub Genes

**Figure 8:**
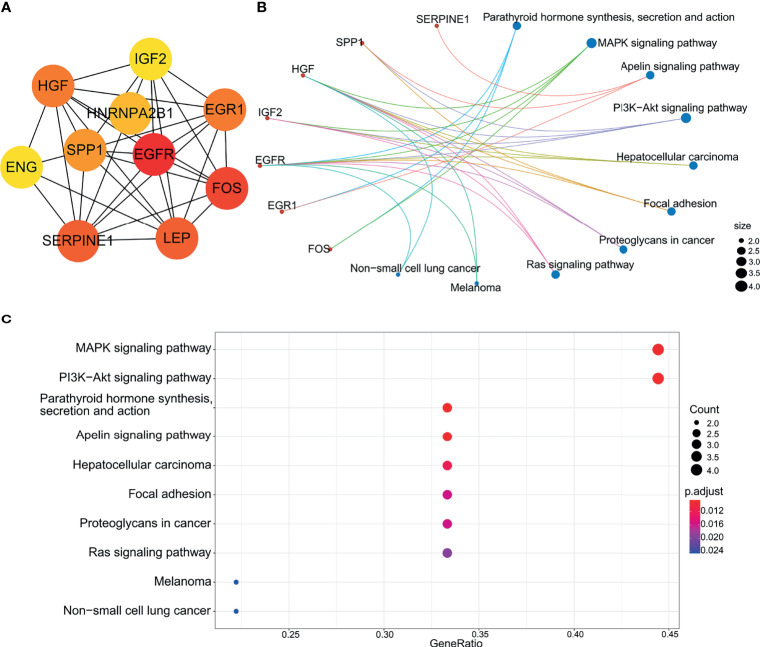
Biological analysis of hub genes. **(A)** The interaction of 10 hub genes. **(B)** and **(C)** the top 10 KEGG enrichment analysis by R language.

Highly-connected genes with a network are considered master regulatory elements otherwise known as hub genes (29). We used the cytoHubba plugin of Cytoscape to reveal the ten most strongly related interactions amongst the DEGs in IVF-ET. This approach generated 10 nodes with 37 edges with the most likely hub genes consisting of *EGFR*, *FOS*, *SERPINE1*, *LEP*, *HGF*, *EGR1*, *SPP1*, *HNRNPA2B1*, *IGF2* and *ENG* (**Figure 8A**). In addition, KEGG analysis of the top 10 enrichment pathways were identified (**Figures 8B, C**).

## Discussion

As one of the main tools of ART, IVF-ET can improve the success rate of infertility treatments for many affected couples. Nonetheless, despite its use for several decades and widespread acceptance, there are still uncertainties associated with IVF-ET in terms of perinatal risks and offspring health. On this basis we hypothesized that complications associated with IVF-ET may be associated with the altered expression of genes that regulate placental development and function. Consequently, we performed genome-wide miRNA and mRNA analyses comparing placentas from IVF-ET assisted and naturally conceived pregnancies. The choice of samples from uncomplicated births was deliberate, as large gene differences from pathological births would be expected while more fundamental changes could be revealed by the experimental design used.

Foremost we considered the epigenetic regulatory mechanisms involving miRNAs as these are recognized effectors of placental-mediated complications during pregnancy (15). Indeed, there has been strong interest in miRNAs as predictive biomarkers for the detection of pathologies in pregnancy with the villous trophoblast being a major source of miRNAs found in maternal circulation (30). Such changes in circulating miRNAs would naturally reflect the changes occurring within the placenta. Our analysis produced a shortlist of 12 miRNAs that were differentially expressed in IVF-ET compared to placental tissue from natural pregnancies.

Three of the 12 differentially expressed miRNAs were confirmed in an independent cohort to be specifically downregulated in IVF-ET placentas, i.e., hsa-miR-204-5p, hsa-miR-1269a and hsa-miR-941. Notably, all three microRNAs have been previously implicated in placental dysfunction and potential fetal growth complications although the underlying mechanisms generally remain unclear. The downregulation of miR-204-5p is associated with fetal growth abnormalities and is enriched in related biological pathways and has also been found to be regulated in the presence of adverse pregnancy-related outcomes (31). For instance, miR-204-5p can inhibit angiogenesis by regulating pro-angiogenic genes such as *ANGPT1* and members of the *VEGF* family (32). miR-1269a was found to be a risk factor for ectopic pregnancy, and currently known risk factors include assisted reproductive technologies such as *in vitro* artificial insemination and hormonal stimulation (33). It has also been suggested that miR-941 is expressed in trophoblast cells and involved in insulin-related intracellular signaling pathways such as Wnt signaling, phosphoinositide-3-kinase, TGF-β signaling, and PPAR-gamma (34). In addition, miR-941 has been shown to target Keap1 to activate the Nrf2 signaling pathway, which in turn protects human endometrial cells from oxygen and glucose deprivation-re-oxygenation induced oxidative stress and programmed necrosis (35). These correlates provide a compelling rationale for the functional significance of these miRNAs in IVF-ET fetal growth.

Given the central importance of miRNA expression in the execution of the transcriptional programs, we predicted transcription factors that might regulate these DEmiRs. The top ranked transcription factor was specificity protein 1 (SP1), a zinc finger transcription factor that binds to a variety of GC-rich motifs and regulates the expression and function of miRNAs as well as the expression of genes associated with embryonic development and differentiation (36, 37). SP1 has been shown to regulate the placental glucocorticoid barrier by repressing the expression of 11β-hydroxysteroid dehydrogenase type 2, leading to fetal growth restriction (FGR) (38). One study identified the interaction of miR-331-3p with SP1 and the interaction of miR-331-3p and miR-1908-5p with glycosyltransferases as a novel mechanism for ABO blood group regulation (39). The second ranked hit in the TF analysis was EGR1 which has been previously implicated in follicular development, ovulation, corpus luteum formation and placental angiogenesis (40), and plays a key role in placental implantation (41). Notably, EGR1 was also identified in the construction of the PPI network.

Here we screened key genes altered in IVF-ET placental tissue based on mRNA next-generation sequencing data and online tools to identify ten hub genes. Among these, EGFR signals through Src- and ERK-mediated pathways activated by VEGFR2. Placental trophoblast cells are enriched in *EGFR* (42) and activation of *EGFR* regulates the proliferation, migration and invasive capacity of extravillous trophoblast cells (43). *FOS* is another molecule involved in angiogenesis. *FOS* belongs to the transcription factor-activated protein 1 (AP-1) superfamily, which is responsible for a variety of cellular processes, including proliferation, differentiation, apoptosis, hypoxia, angiogenesis and steroidogenesis (44, 45), as demonstrated in trophoblast cells (46). *LEP*, an important metabolic hormone, is highly expressed in the placenta and regulates placental, fetal growth and angiogenesis (47). *SPP1*, also called osteopontin (*OPN*), is located in the cytoplasm of placental syncytiotrophoblast and capillary endothelial cells and is considered a marker of placental bed remodeling. Placental development occurs in a hypoxic environment and can stimulate angiogenesis through upregulation of the vascular endothelial growth factor inhibitor of fibrinolytic plasminogen activator 1 (*SERPINE1*) (48).

Considering the important role of these key genes in placental development and angiogenesis, it was instructive to consider how these gene networks were impacted by altered miRNA expression. We used the intersection of miRNA and mRNA sequencing data to construct a microRNA-mRNA network. This analysis revealed more complexity in predicted interactions for the downregulated DeMiRs compared to their upregulated counterparts. Indeed, the downregulated DeMiRs display a far more extensive repertoire of target genes, particularly, hsa-miR-204-5p, hsa-miR-1269a and hsa-miR-941, for which, as discussed above, have tangible functional links to different aspects of placental regulation. Nonetheless, more work is needed to determine which gene targets are most functionally important but further understanding of the underlying mechanisms may help to reduce pregnancy complications and improve offspring safety.

Our current study has its limitations. The discovery cohort was small and we only validated selected miRNAs in the placentas of 8 IVF-ET assisted conception patients and 8 normal controls, which may reduce the reliability of our findings. Moreover, while we were diligent to sex match the placentals and minimize sampling bias by location, placental tissue is known to be divided into maternal, intermediate and fetal zones with different gene expression profiles (49) and inter-placental differences are inevitable. Moreover, the molecular interactions and proposed functional relationships proposed by the bioinformatic analyses need formal verification in both experimental and clinical settings. Thus, more work is needed to explore the specific functions of differentially expressed genes in IVF-ET placentas and their mechanisms.

## Conclusions

In summary, we constructed a miRNA-mRNA regulatory network to regulate the expression of genes essential for IVF-ET placental development and function using bioinformatic analysis. Our data reveal both previously identified miRNAs and mRNAs associated with placental dysfunction and pregnancy complications along with novel candidates. As such these data represent a ready resource for subsequent investigations into the effects of IVF-ET on placental development and function and may provide a basis for future prevention and treatment of adverse perinatal outcomes.

## Data Availability Statement

The data presented in the study is deposited in the NCBI repository, accession number GSE186149.

## Ethics Statement

Tissue collection was approved by the Ethics Committee of the Third Affiliated Hospital of Zhengzhou University. The patients/participants provided their written informed consent to participate in this study. Written informed consent was obtained from the individual(s) for the publication of any potentially identifiable images or data included in this article.

## Author Contributions

SY conducted the majority of the experiments and prepared the manuscript. WZ, MM, and SS were involved in the analysis and interpretation of the data. CY, RZ, NZ, SR, and HW collected samples of placenta and clinical data. RT contributed to the revision and critical discussion of the article. YG was engaged in research design, article drafting and critical discussion. All authors contributed to the article and approved the submitted version.

## Funding

This work was supported by the Henan Province Science and Technology Tackling Plan awarded to YG (Grant no. 202102310061) with further funding support from the State Key Laboratory of Reproductive Medicine, Nanjing Medical University awarded to YG (Grant no. SKLRM-K201902 and SKLRM-K201903).

## Conflict of Interest

The authors declare that the research was conducted in the absence of any commercial or financial relationships that could be construed as a potential conflict of interest.

## Publisher’s Note

All claims expressed in this article are solely those of the authors and do not necessarily represent those of their affiliated organizations, or those of the publisher, the editors and the reviewers. Any product that may be evaluated in this article, or claim that may be made by its manufacturer, is not guaranteed or endorsed by the publisher.
