# Supplementary material for: Integrated Analysis of Hub Genes and MicroRNAs in Human Placental Tissues from In Vitro Fertilization-Embryo Transfer
Source: Front Endocrinol (Lausanne). 2021 Nov 11;12:774997. doi: 10.3389/fendo.2021.774997 (PMC8632620; doi:10.3389/fendo.2021.774997)
Supplement: Supplementary file 1 [file Table_1.docx]

Supplementary Table SI The miRNA-mRNA network

| Gene symbol | *P* Value | LogFC | Up/Down |
| --- | --- | --- | --- |
| ABI3BP | 0.001394876 | 1.452656 | Up |
| AC004528.1 | 0 | -28.7997 | Down |
| AC006262.5 | 0.000286951 | 2.095436 | Up |
| AC008914.1 | 0 | -26.8287 | Down |
| AC025262.1 | 2.2E-27 | -22.9905 | Down |
| AC026407.1 | 0.025457086 | 19.62931 | Up |
| AC073657.1 | 0.014576937 | -19.5093 | Down |
| AC079354.4 | 7.88E-22 | 23.02896 | Up |
| AC092964.1 | 0.014576937 | -19.5754 | Down |
| AC138393.1 | 4.82E-43 | -23.6362 | Down |
| ACP2 | 0.013547451 | 0.990676 | Up |
| ADAM12 | 4.72E-31 | 0.712519 | Up |
| ADIRF-AS1 | 0.003720473 | -0.79271 | Down |
| AFF1 | 0.000235928 | 0.861423 | Up |
| AL050302.1 | 1.43E-15 | 22.54768 | Up |
| AL109927.1 | 0.02718708 | -0.5935 | Down |
| AL161784.1 | 6.25E-08 | -1.32009 | Down |
| AL162431.1 | 5.84E-09 | -21.3777 | Down |
| AL627309.2 | 1.73E-39 | 23.85709 | Up |
| ANGPT2 | 0.000000501 | 1.963656 | Up |
| ANKRD12 | 0.004315587 | 1.296555 | Up |
| AP000758.1 | 0.000000297 | 21.42871 | Up |
| AP001579.1 | 2.27E-36 | -23.3975 | Down |
| ARHGAP26 | 0.023371047 | 1.091109 | Up |
| ARID5B | 0.040473799 | 1.256773 | Up |
| ARL5A | 0.028793672 | 0.807681 | Up |
| ATP6V1C2 | 0.002110169 | 1.081044 | Up |
| BACE2-IT1 | 0.001006931 | 0.616587 | Up |
| BCL6 | 0.001883029 | 1.518702 | Up |
| BCLAF1 | 0.038247163 | 0.84325 | Up |
| BGN | 1.16E-08 | 0.787091 | Up |
| C1GALT1 | 0.003782172 | 0.994783 | Up |
| C6orf89 | 0.047690903 | 0.878015 | Up |
| C9orf78 | 0.030586656 | 1.127078 | Up |
| CALD1 | 6.53E-10 | 1.054395 | Up |
| CASP4 | 0.001614797 | 0.926131 | Up |
| CAST | 0.029806533 | 0.62065 | Up |
| CBR1 | 0.01804652 | 0.874182 | Up |
| CCK | 0.002737909 | -0.88066 | Down |
| CDK11B | 0.001774767 | 0.994983 | Up |
| CDK17 | 0.045855383 | 1.215174 | Up |
| CEACAM1 | 0.038063689 | 1.168341 | Up |
| CGB | 3.1E-16 | -0.77801 | Down |
| CITED4 | 0.002206317 | -0.91737 | Down |
| CLIC4 | 0.000821182 | 0.750357 | Up |
| COBLL1 | 1.32E-08 | 1.001793 | Up |
| COL17A1 | 0.006157705 | 1.296733 | Up |
| COL18A1 | 0.013766116 | -0.77633 | Down |
| CREB3L2 | 0.008709755 | 0.858579 | Up |
| CRHBP | 0.001557632 | -2.16096 | Down |
| CSF2RB | 0.000261582 | 1.158336 | Up |
| CSF3R | 0.000001 | 0.683068 | Up |
| CSHL1 | 4.09E-94 | -0.82458 | Down |
| CTD-2012K14.4 | 0.029160146 | 1.382641 | Up |
| CTD-2228K2.7 | 0.009683339 | 2.49888 | Up |
| CTSF | 0.005371075 | -0.86861 | Down |
| CYP19A1 | 8.28E-44 | 0.732564 | Up |
| CYR61 | 0.021713101 | 0.724308 | Up |
| DAB2 | 0.0000203 | 0.631927 | Up |
| DDX23 | 0.022449874 | 0.957936 | Up |
| DDX3Y | 0.021415688 | -0.96037 | Down |
| DERL3 | 1.57E-13 | 2.547253 | Up |
| DHRS2 | 3.02E-09 | 2.29321 | Up |
| DNASE1L3 | 0.02661446 | -1.29183 | Down |
| DUSP1 | 1.06E-09 | 1.078196 | Up |
| ECM1 | 0.023223536 | 0.766513 | Up |
| EGFR | 0.000000142 | 1.044305 | Up |
| EGR1 | 0.00825414 | 1.059168 | Up |
| EID2 | 0.042222987 | -0.5845 | Down |
| EIF3A | 0.018118605 | 0.828836 | Up |
| EIF4G2 | 1.75E-10 | 0.87024 | Up |
| ENG | 0.000302765 | 0.843373 | Up |
| EPB41L2 | 0.019599639 | 1.044006 | Up |
| ERBB3 | 0.030586656 | 1.108699 | Up |
| ERRFI1 | 3.49E-12 | 1.526004 | Up |
| ERV3-1 | 0.000228697 | 0.785965 | Up |
| ERVW-1 | 0.000134328 | 0.632545 | Up |
| EWSR1 | 0.000569067 | 0.907169 | Up |
| F5 | 0.000320439 | 1.35749 | Up |
| FLT1 | 5.27E-42 | 1.416347 | Up |
| FMR1 | 0.038247163 | 0.827771 | Up |
| FNDC3B | 0.039310125 | 0.793101 | Up |
| FOS | 2.47E-25 | 1.311302 | Up |
| FOSB | 6.57E-09 | 1.739491 | Up |
| FRZB | 3.87E-08 | -1.07638 | Down |
| FSTL3 | 1.81E-26 | 1.774514 | Up |
| GADD45G | 0.001435022 | 0.688592 | Up |
| GBA | 0.000134008 | 1.170303 | Up |
| GCM1 | 0.0000695 | 1.287165 | Up |
| GCSH | 0.002957716 | -0.94883 | Down |
| GJA5 | 0.033433348 | 0.723755 | Up |
| GNB1 | 0.006708618 | 0.785003 | Up |
| GNG11 | 5.18E-09 | -0.60908 | Down |
| GOLGA4 | 0.000826618 | 1.115024 | Up |
| GP6 | 0.028052577 | 2.81909 | Up |
| H19 | 1.48E-28 | 0.598811 | Up |
| HAPLN1 | 0.0149885 | 1.137667 | Up |
| HBA1 | 3.52E-139 | -1.20905 | Down |
| HBA2 | 1.17E-175 | -1.13519 | Down |
| HBG2 | 1.98E-64 | -0.91785 | Down |
| HDLBP | 0.01917305 | 0.667307 | Up |
| HEXB | 5.38E-15 | 1.014854 | Up |
| HGF | 0.031876266 | 0.715165 | Up |
| HLA-C | 0.001887518 | 0.823017 | Up |
| HLA-DRB1 | 0.044028741 | -0.7813 | Down |
| HLA-G | 0.000000531 | -1.54691 | Down |
| HMGN3 | 0.038372122 | 0.618725 | Up |
| HMSD | 0.000000492 | -7.68662 | Down |
| HNRNPA2B1 | 0.0000356 | 0.605658 | Up |
| HNRNPD | 0.0000853 | 0.717791 | Up |
| HNRNPH3 | 0.00687706 | 0.751324 | Up |
| HP1BP3 | 0.015731858 | 0.610677 | Up |
| HPGD | 7.71E-27 | 1.297413 | Up |
| ID1 | 0.0000439 | -0.72219 | Down |
| IGF2 | 0.00000001 | 0.601114 | Up |
| IGFBP1 | 0.005436578 | 0.662876 | Up |
| IL1RAP | 0.008874742 | 0.917501 | Up |
| INHBA | 7.72E-17 | 1.494363 | Up |
| INSIG1 | 0.016429407 | 0.73723 | Up |
| ITGA5 | 0.039385667 | 0.594813 | Up |
| JUp | 0.021564839 | 0.709929 | Up |
| KIF2A | 0.009708711 | 0.855579 | Up |
| KLF4 | 0.027481758 | 0.87607 | Up |
| KLF5 | 0.005673525 | 1.202576 | Up |
| KRTAP26-1 | 0.040473799 | 1.346672 | Up |
| LAIR2 | 0.000000466 | -3.70789 | Down |
| LAMC1 | 0.002948806 | 0.734785 | Up |
| LDHA | 0.037287334 | 0.665527 | Up |
| LEP | 3.84E-46 | 3.81517 | Up |
| LEPREL1 | 0.042032716 | 1.389219 | Up |
| LIFR | 0.000431687 | 1.081213 | Up |
| LIMCH1 | 0.003188171 | 1.182099 | Up |
| LIMS3 | 0.024692905 | 1.195199 | Up |
| LUC7L3 | 0.00010101 | 1.045035 | Up |
| LUZP6 | 0.0000713 | -2.78055 | Down |
| LYNX1 | 0.036616482 | 2.019362 | Up |
| LYZ | 0.005688031 | -0.84358 | Down |
| MALAT1 | 1.65E-24 | 1.451366 | Up |
| MLL5 | 0.035886183 | 1.197816 | Up |
| MME | 0.040432743 | 0.875825 | Up |
| MPHOSPH8 | 0.023453403 | 1.223257 | Up |
| MT-ATP8 | 0 | 1.237571 | Up |
| MTRNR2L12 | 0.010599604 | 1.816591 | Up |
| MTSS1L | 0.001394876 | 1.404385 | Up |
| MUC15 | 5.03E-20 | 1.474689 | Up |
| MXRA7 | 0.006826842 | -0.66116 | Down |
| MYADM | 0.000393775 | 1.106866 | Up |
| MYZAP | 0.008246209 | 3.40074 | Up |
| NARS | 0.011610698 | 1.029489 | Up |
| NCL | 0.00459213 | 0.737563 | Up |
| NDEL1 | 0.041842885 | 0.805836 | Up |
| NDRG1 | 0.00000996 | 1.219549 | Up |
| NEAT1 | 1.21E-80 | 0.896277 | Up |
| NFE2L1 | 0.000194053 | 0.992026 | Up |
| NFYC | 0.019485244 | 1.011955 | Up |
| NME2 | 0.00000126 | -3.05744 | Down |
| NSRP1 | 0.000179506 | 1.70224 | Up |
| NUCB2 | 0.001910509 | 0.732405 | Up |
| PAPPA | 4.28E-12 | 0.654792 | Up |
| PAPPA2 | 5.78E-23 | 1.224718 | Up |
| PAPPA-AS1 | 5.25E-92 | 1.008104 | Up |
| PDCD4 | 0.00423288 | 0.716566 | Up |
| PGF | 4.72E-17 | -0.8465 | Down |
| PHACTR2 | 0.023229788 | 0.935794 | Up |
| PLA2G2A | 0.000770227 | -0.62513 | Down |
| POR | 0.000370721 | 1.004733 | Up |
| PPP1R12A | 0.023371047 | 1.101821 | Up |
| PPP1R13L | 0.000129554 | 1.063266 | Up |
| PPP1R14A | 0.014009677 | -0.65999 | Down |
| PROCR | 0.04802951 | 0.743848 | Up |
| PROSER1 | 0.012608263 | 0.969203 | Up |
| PRRC2C | 0.019599639 | 1.0396 | Up |
| PSG11 | 3.18E-23 | 0.874204 | Up |
| PSIP1 | 0.010763312 | 1.223554 | Up |
| PVRL3 | 0.038578641 | 0.606389 | Up |
| PVRL4 | 0.044587717 | 0.748196 | Up |
| RAC1 | 0.000172822 | 0.815625 | Up |
| RAP1B | 0.000875775 | 0.779293 | Up |
| RBM25 | 0.028286918 | 0.802817 | Up |
| RBM5 | 0.007925476 | 0.791574 | Up |
| RBP4 | 0.042568585 | -1.13065 | Down |
| RN7SL1 | 0.000000225 | 0.913974 | Up |
| RP11-10A14.3 | 0.04165196 | 1.681227 | Up |
| RP11-166B2.1 | 0.01778529 | 0.927141 | Up |
| RP11-264B14.2 | 0.019878323 | -0.86715 | Down |
| RP11-286N22.8 | 0.005833132 | 2.242784 | Up |
| RP11-298J23.9 | 0 | 27.78552 | Up |
| RP11-33B1.4 | 0.000825783 | 2.382122 | Up |
| RP11-366K18.3 | 0.008442557 | 0.6411 | Up |
| RP11-55L4.1 | 0.023610243 | -1.67614 | Down |
| RP11-612B6.2 | 0.008246209 | 20.02352 | Up |
| RP11-76E12.1 | 0.026167165 | -0.94943 | Down |
| RP11-860B13.3 | 0.000259522 | -20.3935 | Down |
| RP11-861E21.1 | 0.044482192 | 19.51287 | Up |
| RP11-9M16.2 | 0.001494316 | 20.38972 | Up |
| RP1-56K13.3 | 0.017163732 | 1.636533 | Up |
| RP1-65P5.3 | 3.34E-46 | -1.7502 | Down |
| RP4-669L17.10 | 0.000110378 | 1.053665 | Up |
| RP5-890E16.4 | 0.0000701 | -1.70142 | Down |
| RPS4Y1 | 0.000299896 | -0.88474 | Down |
| RRBP1 | 0.003047602 | 0.729722 | Up |
| SCIN | 0.00000934 | 1.120496 | Up |
| SEC62 | 0.0059273 | 0.793732 | Up |
| SEMA6A | 0.002970884 | 0.944382 | Up |
| SERPINE1 | 9.13E-10 | 1.031252 | Up |
| SH3BP5 | 0.0000013 | 1.653806 | Up |
| SIL1 | 0.002142321 | 0.938331 | Up |
| SLC38A1 | 0.038012476 | 0.631944 | Up |
| SLC7A8 | 0.039310125 | 0.776614 | Up |
| SLCO2A1 | 0.000113235 | 1.213818 | Up |
| SNAP23 | 0.000168681 | 0.923171 | Up |
| SOCS1 | 0.006314634 | 1.484971 | Up |
| SOX18 | 0.011239126 | -0.90252 | Down |
| SPP1 | 5.9E-18 | 0.954565 | Up |
| SPTLC3 | 1.42E-12 | 1.324445 | Up |
| SRP72 | 0.000428248 | 1.277803 | Up |
| SRRM2 | 0.000961103 | 0.799555 | Up |
| SRSF11 | 0.00239854 | 0.748502 | Up |
| STAG2 | 0.000272298 | 1.358195 | Up |
| STS | 1.73E-09 | 1.296366 | Up |
| SUB1 | 0.038218679 | 0.704281 | Up |
| SUpT5H | 0.000636977 | 1.214663 | Up |
| SYF2 | 0.049632195 | 0.694636 | Up |
| SYNCRIP | 0.010064765 | 0.897523 | Up |
| TAB3-AS2 | 6.74E-12 | 3.324193 | Up |
| TAC3 | 3E-19 | -1.09625 | Down |
| TACC2 | 0.000149249 | 1.13729 | Up |
| TAF15 | 0.0102192 | 0.86187 | Up |
| TCEAL4 | 0.024276527 | 0.796953 | Up |
| TCL6 | 0.005238607 | 0.747618 | Up |
| TFAP2A | 0.011212016 | 0.628277 | Up |
| TFPI | 5.96E-34 | 1.102171 | Up |
| THY1 | 0.032582219 | 0.874116 | Up |
| TINCR | 0.025944168 | 0.719672 | Up |
| TLR3 | 0.000038 | 1.598957 | Up |
| TMEM54 | 2.37E-08 | -0.85695 | Down |
| TOP1 | 0.007810676 | 0.948424 | Up |
| TPBG | 0.026548401 | 1.260626 | Up |
| TPP1 | 0.004863236 | 0.753002 | Up |
| TPRXL | 0.042910633 | 1.176059 | Up |
| TRIQK | 0.017424716 | 1.443517 | Up |
| TSC22D1 | 0.000383273 | 0.81331 | Up |
| TSC22D3 | 0.001799437 | 0.996386 | Up |
| UBAP2L | 0.004907882 | 0.889217 | Up |
| UBE2V1 | 0.018997291 | 0.819017 | Up |
| UCA1 | 0.000432317 | 1.50454 | Up |
| UCK2 | 0.000175476 | -0.91177 | Down |
| VAMP5 | 0.032262192 | -0.67856 | Down |
| VAV3 | 0.006449415 | 0.851584 | Up |
| VGLL3 | 0.000130343 | 0.715652 | Up |
| VPS4B | 0.023415352 | 1.022032 | Up |
| WAC | 0.0000454 | 1.11964 | Up |
| XIST | 1.33E-08 | 1.539443 | Up |
| XPO7 | 0.040128095 | 1.045737 | Up |
| ZFAND3 | 0.046850781 | 0.794868 | Up |
| ZFAT | 1.04E-08 | 1.394842 | Up |
| ZNF595 | 0.000453961 | 1.237711 | Up |
| ZNF888 | 0.008136679 | 0.616165 | Up |
